# Self-Assembly and Headgroup Effect in Nanostructured Organogels via Cationic Amphiphile-Graphene Oxide Composites

**DOI:** 10.1371/journal.pone.0101620

**Published:** 2014-07-01

**Authors:** Tifeng Jiao, Yujin Wang, Qingrui Zhang, Xuehai Yan, Xiaoqing Zhao, Jingxin Zhou, Faming Gao

**Affiliations:** 1 Hebei Key Laboratory of Applied Chemistry, School of Environmental and Chemical Engineering, Yanshan University, Qinhuangdao, China; 2 National Key Laboratory of Biochemical Engineering, Institute of Process Engineering, Chinese Academy of Sciences, Beijing, China; RMIT University, Australia

## Abstract

Self-assembly of hierarchical graphene oxide (GO)-based nanomaterials with novel functions has received a great deal of attentions. In this study, nanostructured organogels based on cationic amphiphile-GO composites were prepared. The gelation behaviors of amphiphile-GO composites in organic solvents can be regulated by changing the headgroups of amphiphiles. Ammonium substituted headgroup in molecular structures in present self-assembled composites is more favorable for the gelation in comparison to pyridinium headgroup. A possible mechanism for headgroup effects on self-assembly and as-prepared nanostructures is proposed. It is believed that the present amphiphile-GO self-assembled system will provide an alternative platform for the design of new GO nanomaterials and soft matters.

## Introduction

Graphene, an atom thick graphite sheet, has received a great deal of attentions because of its unique electronic, thermal and mechanical properties etc. [1]–[12]. Recent works have demonstrated that self-assembly is a powerful technique for constructing hierarchical graphene oxide (GO)-based nanomaterials with novel functions [13]–[20]. In particular, graphene oxide can easily be functionalized through both covalent and non-covalent bonding; thereby making graphene oxide an important building block for the synthesis of new materials [21]–[25].

Since their reports by Zu et al. [26] and Vickery et al. [27] in 2009, polymer-GO hydrogels have attracted considerable attentions [28], [29]. Recently, GO-based hydrogels have been investigated by several groups for the purpose of producing 2D macro-assemblies, which have a variety of possible applications including supercapacitors [30], [31], drug-release [32], tissue scaffolds [33], and adsorbents [34]. For example, the Shi and his coworkers have recently developed self-assembled graphene hydrogels with 3D networks by a convenient one-step hydrothermal method or chemical reduction [35], [36]. They have also prepared graphene oxide/DNA composite gels and graphene oxide/haemoglobin composite gels [37]. The hydrogels have strong mechanical strength, high electrical conductivity, excellent thermal stability and large pore size. However, GO is merely incorporated into hydrogels disorderly and randomly in the above-mentioned cases. Compared with the hydrogels formed in water, GO composite organogels entrapped expected organic solvents have some advantages in some aspects e.g., the fabrication of electrode materials or supercapacitors without addition of polymer binders or conducting additives [38]. At present, there are few reports on the gelation of common organic solvents by GO-amphiphile composites, but some ionic liquids [39], [40], propylene carbonate [41], and pyrene-containing peptides [42], [43].

In this study, we have demonstrated the formation of nanostructured organogels based on self-assembly of cationic amphiphile-GO composites in common organic solvents. Three used cationic compounds contained different ammonium or pyridinium headgroups in molecular structures, which showed distinct π-π stacking and spatial hindrance in self-assembly process. Interestingly, it is found that nanostructures differ in different organic solvents as evidenced by morphological and spectral studies. The diversity of nanostructures in the organogels results from the variation of headgroups of amphiphiles coupled to GO. Furthermore, the mechanism associated with nanostructural formation is proposed. Soft matters fabricated through the gelation of amphiphile-GO might offer a good alternative for design and development of GO-based materials and devices.

## Experimental Methods

### 1. Materials

The starting materials, 1-bromohexadecane, 4,4'-dipyridyl, and hexadecyl trimethyl ammonium bromide (abbreviated as CTAB, product number A15235, purity 98%) were purchased from Alfa Aesar (Tianjin) chemical Co., Ltd. (Beijing, China). Cetylpyridinium bromide (abbreviated as C16Py) with analysis purity were purchased from Sinopharm Chemical Reagent Beijing CO., Ltd., China. The solvents were obtained from Beijing Chemicals and were distilled before use. Deionized water was used in all cases.1,1'-Dihexadecyl-4,4'-bipyridiniumbromide (abbreviated as BPy) was synthesized in our laboratory by reacting 4,4'-dipyridyl with 1-bromohexadecane. Simply speaking, the 1-bromohexadecane and 4,4'-dipyridyl were heated in dried ethanol for 2 days at 78°C. After that, the reaction mixtures were cooled and evaporated to dryness. Then, the residues were purified by recrystallization in ethanol solution as a yellow solid. The final product was confirmed by ^1^H NMR and elemental analysis.

### 2. Preparation of graphene oxide (GO)

The GO sheets were prepared according to the method described by Hummer [44] with some modification [45], specifically as follows: graphite, NaNO_3_, concentrated H_2_SO_4_ mixing together in an ice bath for 30 min, followed by slow addition of KMnO_4_. The reaction mixture was stirred at 35°C for 2 h, and then the temperature was slowly raised to 60°C during the other 2 h. The mixture was then added to water and was stirred at 90°C for 5 h, adding 30% H_2_O_2_, and then filtering. For purification, the product was washed with 5% of HCl and then DI H_2_O for several times. The filter cake was dissolved in water, the graphene oxide flakes can be obtained by centrifugation. Finally the product was 60°C dried in a vacuum for 12 h.

### 3. Gelation test

A weighted amount of amphiphiles and GO (10 mg/mL) in selected pure organic solvent were placed into a sealed glass bottle and ultrasonic dispersed evenly for 20 min. Then the solution was heated in a water bath at 70°C for 20 min. After that, the solution was cooled to room temperature in air and the test bottle was inversed to see if a gel was formed. When the binary mixture formed a gel by immobilizing the solvent at this stage, it was denoted as ‘G’. The system, in which the potential gelator could not be dissolved, was designated as an insoluble system (I). Experimental details are listed in Table 1.

**Table 1:** Gelation properties of three amphiphiles-GO composites at room temperature.

Solvents	C16Py-GO	BPy-GO	CTAB-GO
DMF	G(5.0)	G(5.0)	G(3.0)
Acetonitrile	I	I	I
Ethanol	I	I	I
n-Propanol	I	I	I
n-Butanol	I	I	I
n-Pentanol	I	I	I
Isopropanol	I	I	I
Isoamyl alcohol	I	I	I
Cyclopentanone	I	G(5.0)	G(3.0)
Cyclohexanone	I	I	G(3.0)
Benzene	I	I	I
Toluene	I	I	I
Nitrobenzene	I	I	I
Aniline	I	I	I
Ethyl acetate	I	I	I
n-Butyl acrylate	I	I	I
n-Hexane	I	I	I
1,4-Dioxane	I	I	G(3.0)
THF	G(3.0)	G(3.0)	G(3.0)
Pyridine	G(3.0)	I	I

DMF, dimethylformamide; THF, tetrahydrofuran; G, gel; I, insoluble; for gels, the critical gelation concentrations of amphiphiles with GO (10 mg/mL) at room temperature are shown in parentheses, [%(w/v)].

### 4. Characterizations

Firstly, the various xerogels were prepared by freeze-drying method with −55°C low temperature through a lyophilizer (FD-1C-50, Beijing Boyikang Experimental Instrument Co., Ltd., China) to remove different organic solvents over 30 h, which could maintain the original nanostructures. Before scanning electron microscope (SEM) measurement, the samples were coated with copper foil fixed by a conductive adhesive tape and shielded with gold. SEM pictures of the xerogel were taken using a Hitachi S-4800 field emission scanning electron microscope (Chiyoda-ku, Japan) with the accelerating voltage of 5–15 kV. All transmission electron microscope (TEM) measurements were carried out on HT7700 equipment (Hitachi, Tokyo, Japan) with 300 mesh copper grids covered with thin amorphous carbon films for analysis. Atomic force microscope (AFM) images were recorded using a multimode 8 scanning probe microscope (Veeco Instrument, Plainview, NY, USA) with silicon cantilever probes. All AFM images were shown in the height mode without any image processing except flattening. The X-ray diffraction (XRD) measurement was conducted using a Rigaku D/max 2550PC diffractometer (Rigaku Inc., Tokyo, Japan). The XRD pattern was obtained using Cu Kα radiation with an incident wavelength of 0.1542 nm under a voltage of 40 kV and a current of 200 mA. The scan rate was 0.5° min^−1^.Transmission Fourier transform infrared (FT-IR) spectra of the xerogel were obtained using a Nicolet iS10 FT-IR spectrophotometer from Thermo Fisher Scientific Inc. (Waltham, MA, USA) with an average of 16 scans and at a resolution of 4 cm^−1^. Thermogravimetry-differential scanning calorimetry (TG-DSC) analyses of the samples were conducted in air condition by using NETZSCH STA 409 PC Luxxsi multaneous thermal analyzer (Netzsch Instruments Manufacturing Co., Ltd., Germany).

## Results and Discussion

**Figure 1:**
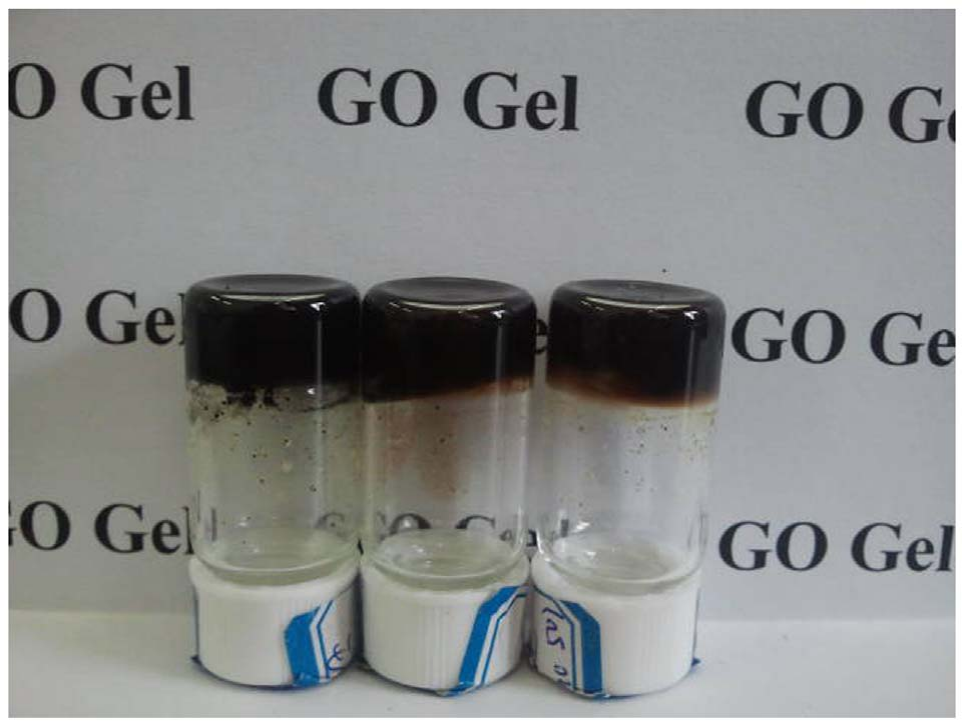
Photograph of organogels of BPy-GO in solvents of DMF, cyclopentanone, and THF (from left to right).

Firstly, the gelation performances of all composites in 20 organic solvents were tested, with the details listed in Table 1. Examination of the table reveals that all of present amphiphile-GO composites are efficient gelators. C16Py-GO composite can gel in solvents of DMF, THF, and pyridine. As for BPy-GO composite, the gelation was observed in DMF, cyclopentanone, and THF. In addition, CTAB-GO composite can form gels in five kinds of solvents such as DMF, cyclopentanone, cyclohexanone, 1,4-dioxane, and THF. Interestingly, it should be mentioned that the three amphiphiles-GO composites with lower concentration or GO alone cannot gel in any present solvents. From the photograph of organogels based on BPy-GO composite in different solvents shown in Figure 1, it is clear that the present as-formed black gels are opaque. It is interesting to note that all present composites can form organogels in solvents of DMF and THF. The data shown in Table 1 indicate that the changes of headgroups in molecular skeletons of cationic amphiphiles can have a profound effect on the gelation of present composites. It seems that the ammonium substituted headgroup in molecular skeletons is more favorable for the gelation of organic solvents in comparison to pyridinium headgroup.

**Figure 2:**
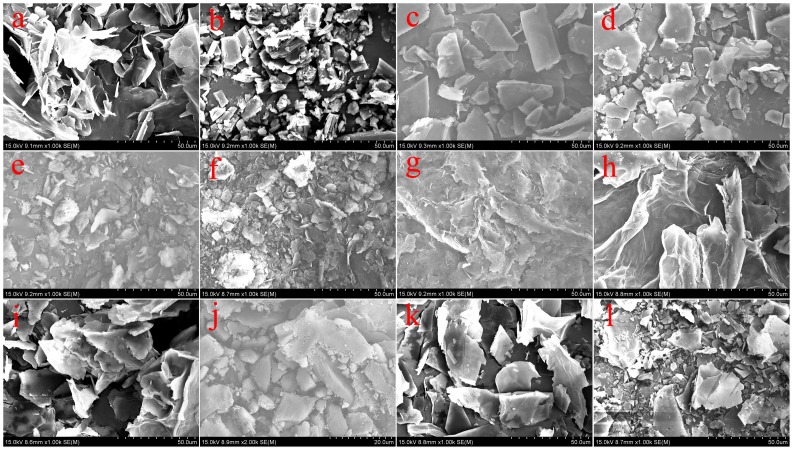
SEM images of xerogels. GO sheets (a), C16Py-GO gels ((b) DMF, (c) THF, and (d) pyridine), BPy-GO gels ((e) DMF, (f) cyclopentanone, and (g) THF), and CTAB-GO gels ((h) DMF, (i) cyclopentanone, (j) cyclohexanone, (k) 1,4-dioxane, and (l) THF).

**Figure 3:**
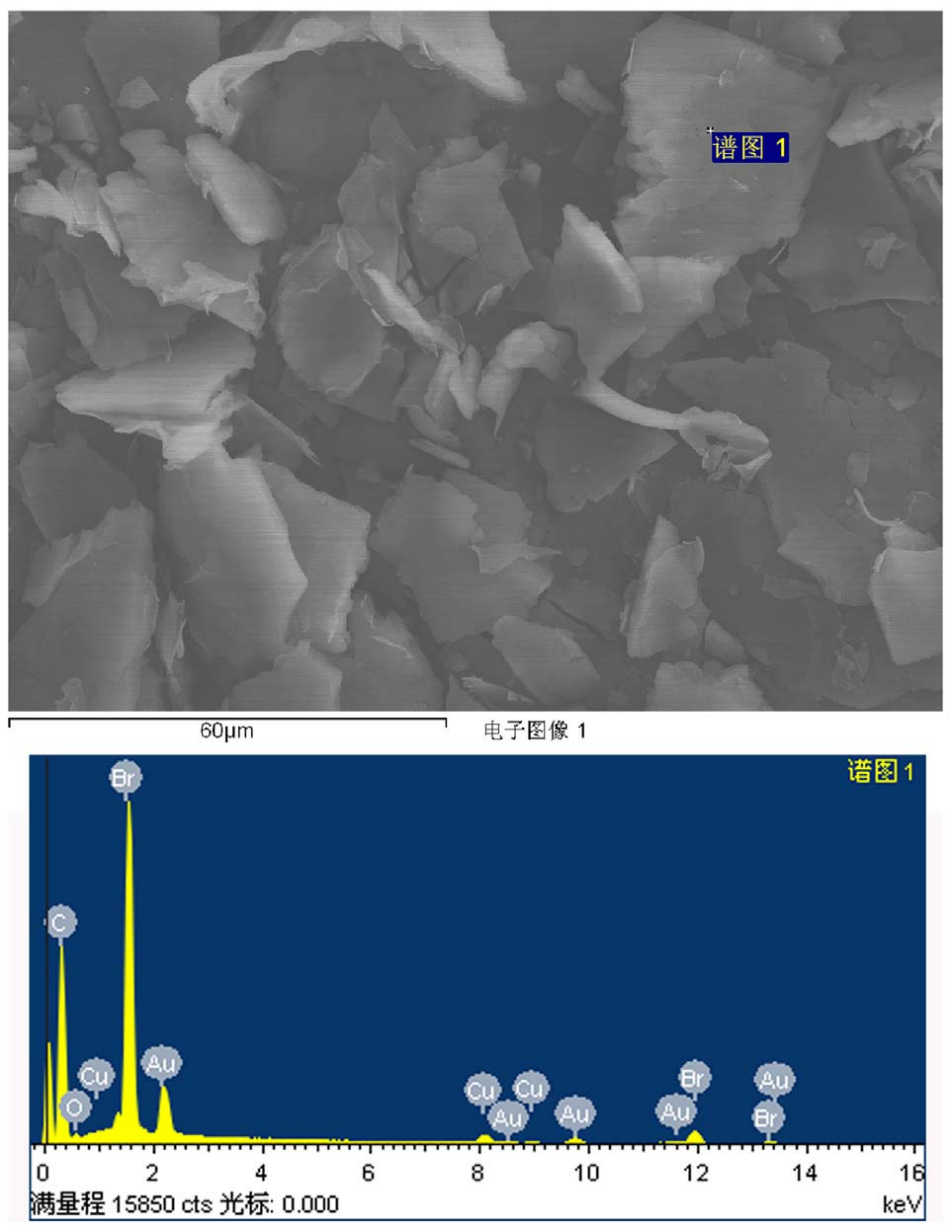
Typical EDXS of xerogels originate from CTAB-GO gels in cyclopentanone. The Cu and Au peaks originate from the substrate of copper foil and the coated gold nanoparticles.

**Figure 4:**
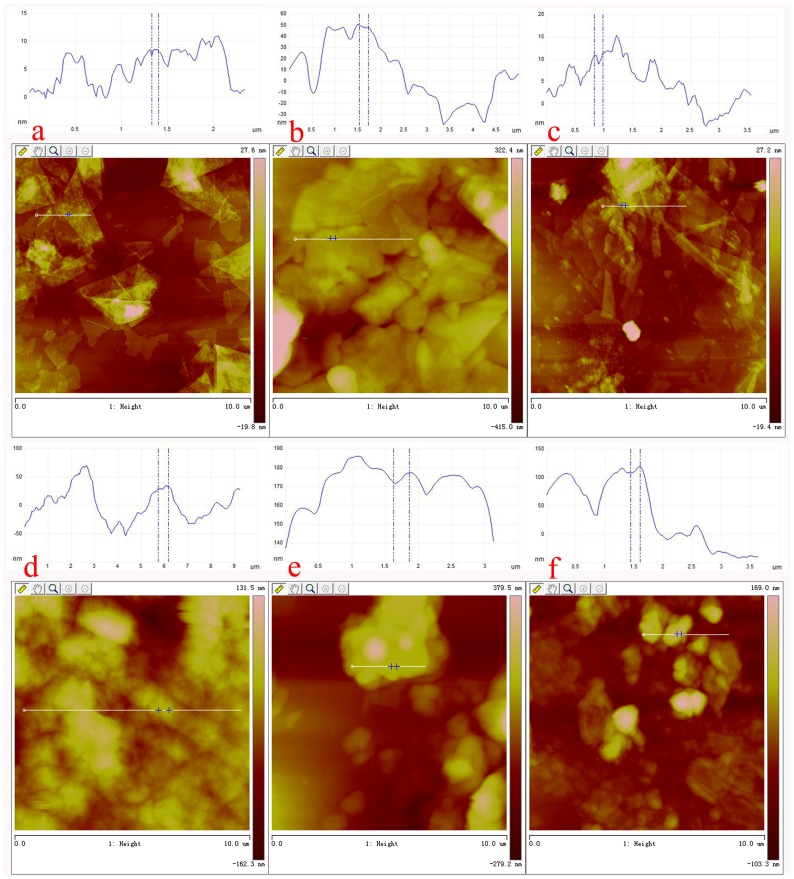
AFM images with section analysis of xerogels. C16Py-GO gels, BPy-GO gels, and CTAB-GO gels in DMF (a, b, and c), and THF (d, e, and f). Scale bars: 10 µm.

**Figure 5:**
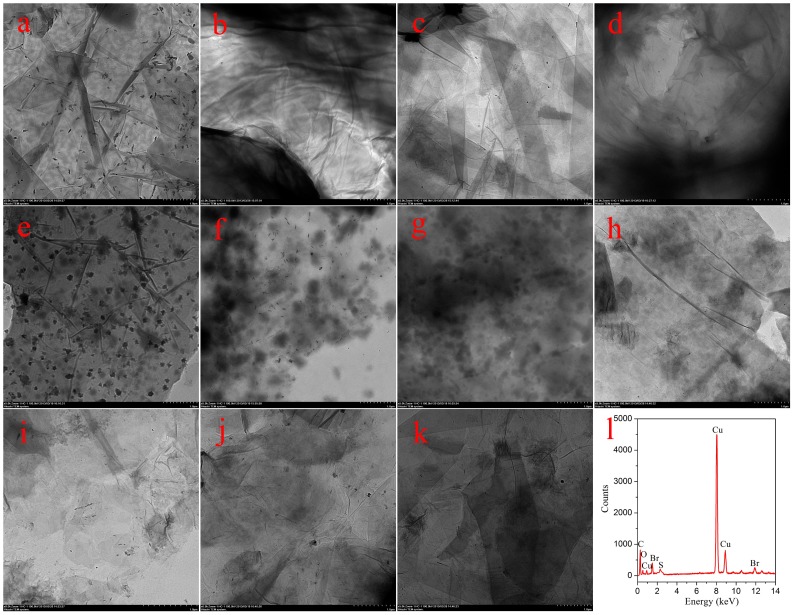
TEM images and typical EDXS (l) of xerogels. C16Py-GO gels ((a) DMF, (b) THF, and (c) pyridine), BPy-GO gels ((d) DMF, (e) cyclopentanone, and (f) THF), and CTAB-GO gels ((g) DMF, (h) cyclopentanone, (i) cyclohexanone, (j) 1,4-dioxane, and (k) THF).Scale bars: 1 µm. The Cu peaks originate from the TEM grid.

With the purpose of obtaining a visual insight into the gel microstructures, the typical nanostructures of the xerogels were studied using SEM. As shown in Figure 2, it is easily observed that the microstructures of the xerogels of all composites in different solvents are significantly different from each other, and the morphologies of the self-assembled aggregates change from wrinkle, lamella, and belt to fiber with the change of solvents. The difference of morphologies is mainly attributed to the various organization modes upon interactive forces between gelators and solvent molecules. In Figure 3, a typical energy dispersive X-ray spectrum (EDXS) of xerogels from CTAB-GO gels shows strong peaks of carbon element originated from GO and amphiphiles as well as bromide element only from amphiphiles, suggesting the combination of GO and amphiphiles in composites. In addition, the morphologies of xerogels of these composites in DMF and THF were investigated by AFM technique. Different aggregates apart from GO sheets were observed in the AFM images as shown in Figure 4, suggesting various self-assembly modes in the organized stacking composites. This may be assigned to the effect of headgroups in molecular skeletons on gelation. Here, it should be mentioned that the thickness of a GO sheet is in the range of 0.5–1.0 nm, larger than the theoretical value of graphene layer (0.34 nm). This is mainly due to the abundant oxygen-containing groups (hydroxyl and epoxy groups) remaining on the surface of the GO sheets [46], [47]. Besides, TEM images of present composites in xerogels state were also investigated, as shown in Figure 5. From these pictures, different aggregates with various sizes and shapes were observed. The morphologies of the aggregates shown in the above images may be rationalized by considering a commonly accepted idea that highly directional intermolecular interactions between amphiphiles and GO, such as electronic interaction or π-π interactions, favor formation of such organized nanostructures [48], [49].

**Figure 6:**
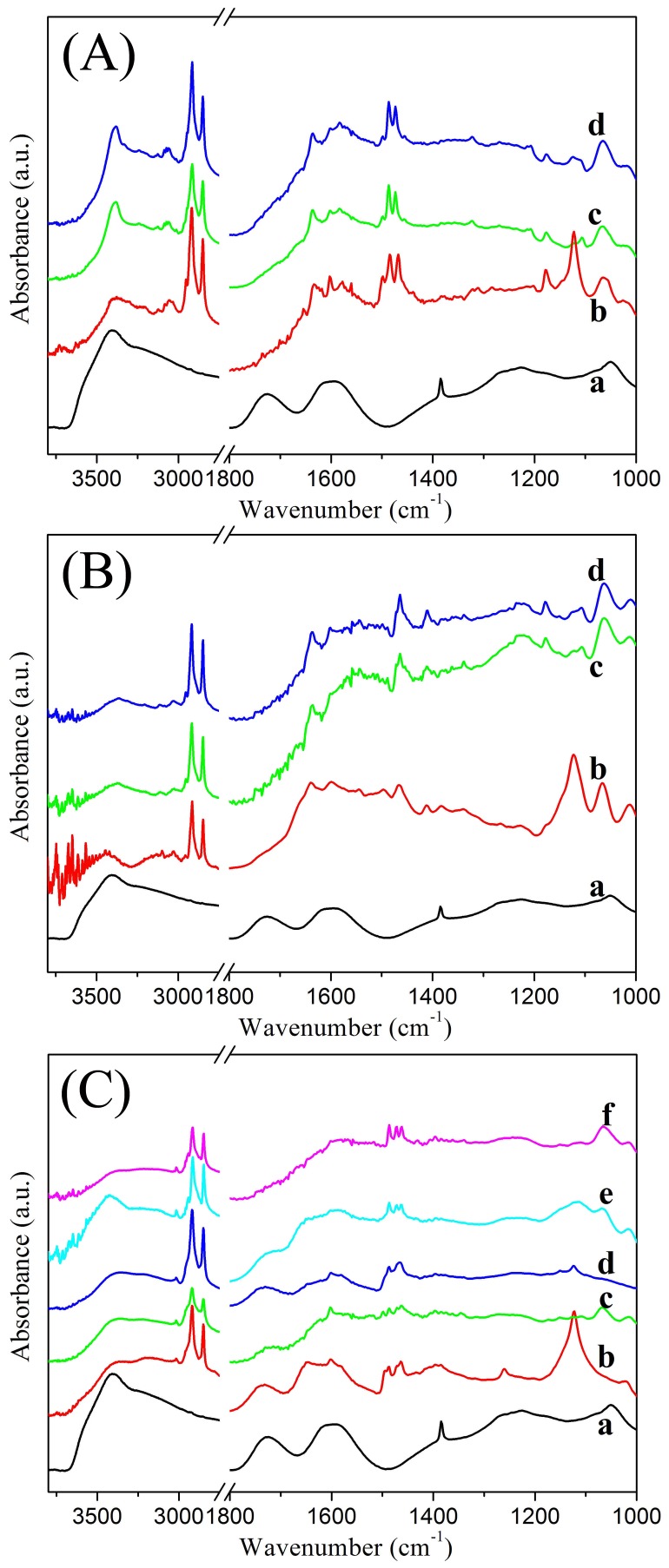
FT-IR spectra of xerogels. (A) GO in KBr pellet (a) and C16Py-GO gels in DMF (b), THF (c), and pyridine (d); (B) GO (a) and BPy-GO gels in DMF (b), cyclopentanone (c), and THF (d); (C) GO (a) and CTAB-GO gels in DMF (b), cyclopentanone (c), cyclohexanone (d), 1,4-dioxane (e), and THF (f).

The FT-IR spectra of xerogels of amphiphile-GO composites and GO sheets in KBr pellet are measured, as shown in Figure 6. The spectrum of the GO indicates the presence of C-O bonds at 1051 cm^−1^, C-O-C bonds at 1265 cm^−1^, C-OH bonds at 1385 cm^−1^, C = O bonds in carboxylic acid and carbonyl at 1724 cm^−1^, -OH bonds at 3402 cm^−1^ [50]. In addition, the peak at 1598 cm^−1^ may result from a skeletal vibration of functional graphitic domains [51]. In addition, as for the xerogels of amphiphile-GO composites, the spectra show composite curves of GO and amphiphiles. As for CTAB-GO gels, the curve shows the presence of -OH bonds at 3375 cm^−1^, CH_2_ stretching vibration bonds at 2918 and 2848 cm^−1^, CH_2_ scissoring vibration bond at 1468 cm^−1^, C = O stretching vibration bonds around 1728 cm^−1^, and C-O bonds at 1122 cm^−1^. The obvious shift of -OH bonds suggested the formation of hydrogen bonding in composites. For the cases of C16Py-GO and BPy-GO gels, obvious change is the disappearance of C = O stretching vibration bonds, which is probably due to the π-π interaction between the GO layers and aromatic pyridinium headgroup [52]. Thus, the present FT-IR results reveal the presence of amphiphiles and GO in the composites as well as the successful combination of both components. In comparison with curve of GO, the band shift of hydroxyl groups in the xerogels of amphiphile-GO composite is caused by the van der Waals force and the hydrogen-bonds between the amphiphiles and GO [53].

**Figure 7:**
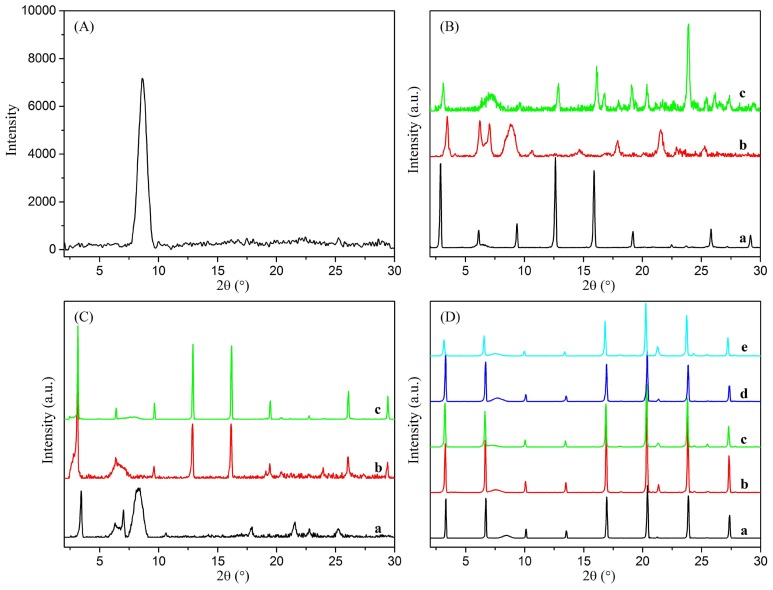
XRD patterns of xerogels. (A) GO sheets; (B) C16Py-GO gels in DMF (a), THF (b), and pyridine (c); (C) BPy-GO gels in DMF (a), cyclopentanone (b), and THF (c); (D) CTAB-GO gels in DMF (a), cyclopentanone (b), cyclohexanone (c), 1,4-dioxane (d), and THF (e).

Besides, with the purpose of investigating the orderly stacking of xerogel nanostructures, the xerogels structures of present composites were investigated by X-ray diffraction (XRD) measurements. The XRD patterns of GO sheets and amphiphile-GO composites are given in Figure 7. The X-ray pattern of GO displays the presence of a strong peak at 2θ = 8.70° corresponding to (001) reflection peak with a layer distance of 1.02 nm [54]. In addition, the three pure amphiphiles exhibit no obvious diffraction peaks, indicating that the present cationic amphiphiles are amorphous on themselves. The curves for samples of CTAB-GO xerogels show similar main peaks in the angle region (2θ values: 3.32°, 6.72°, 10.08°, 13.50°, 16.94°, 20.40°, 23.86°, and 27.34°) corresponding to the *d* values of 2.66, 1.32, 0.88, 0.66, 0.52, 0.44, 0.37, and 0.33 nm. The corresponding *d* values follow a ratio of 1∶1/2∶1/3∶1/4∶1/5∶1/6∶1/7, suggesting a good lamellar structure of the aggregates in the gel [48]. For C16Py-GO and BPy-GO composites, weaker peaks were obtained with significant background noise, suggesting a polycrystalline or amorphous structure in xerogels. The present XRD results described above demonstrate again that the substituent headgroups had great effect on the assembly modes of the compounds.

**Figure 8:**
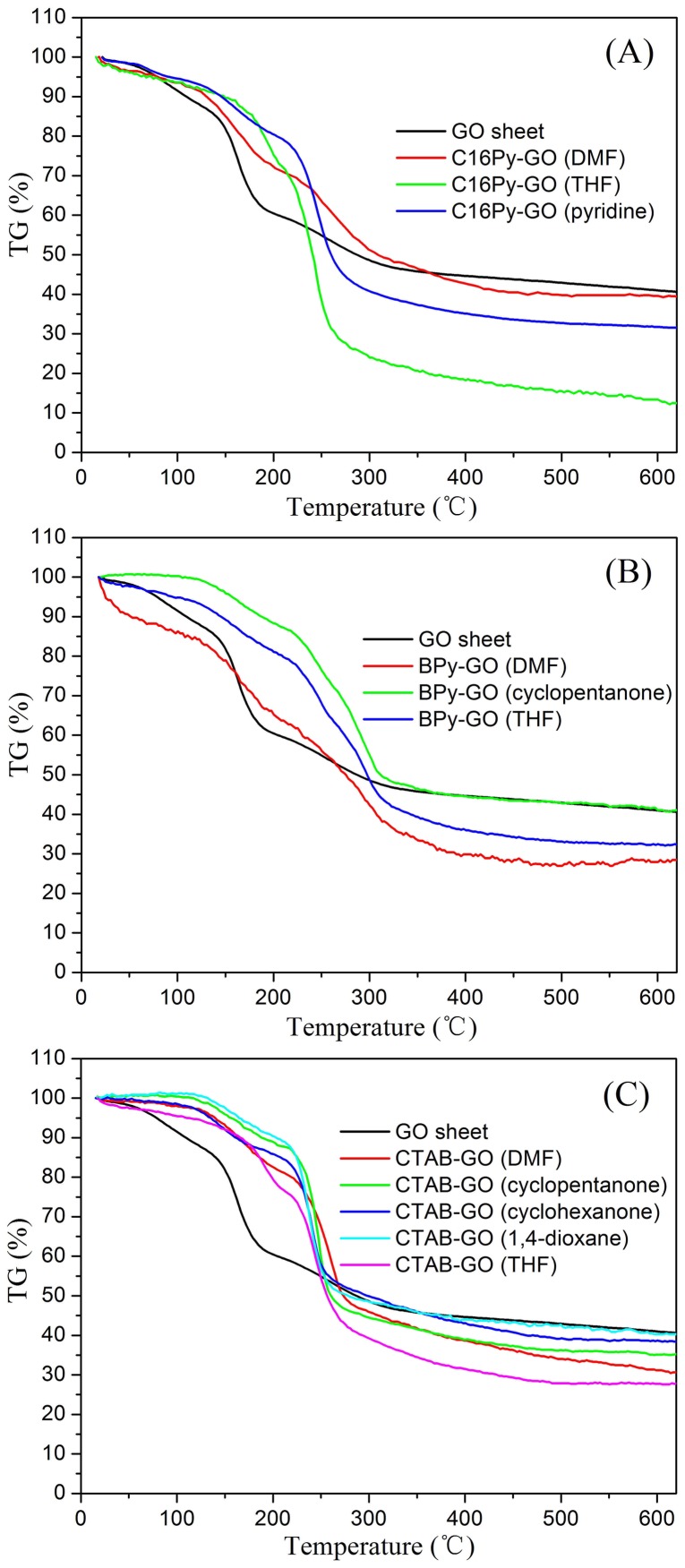
TG curves of xerogels. (A) GO sheet and C16Py-GO gels in DMF, THF, and pyridine; (B) GO sheet and BPy-GO gels in DMF, cyclopentanone, and THF; (C) GO sheet and CTAB-GO gels in DMF, cyclopentanone, cyclohexanone, 1,4-dioxane, and THF.

The thermal stability of GO sheet and amphiphile-GO composites are also investigated by simultaneous thermal analysis (TG-DSC). The TG curves of GO sheet and xerogels of composites are measured, as shown in Figure 8, with respective data summarized in Table 2. In case of GO, major weight loss at the temperatures below 150°C and range of 150–300°C is attributed to the removal of residual water and most of the labile oxygen-containing functional groups. The 41% residual weight of GO indicates that some functional groups exist in GO surface before the thermal treatment. For amphiphile-GO composites, incorporation of amphiphiles in the interface of GO sheets decreased the thermal stability of the composites. Although the thermal degradation process is similar, the major degradation in the composites start at lower temperatures compared to pristine GO sheets as shown in the Table 2. In the TGA curve of amphiphile-GO composites, the weight loss near 200°C can be attributed to pyrolysis of the labile oxygen-containing functional groups. After 200°C, major weight loss had occurred, presumably due to the decomposition of the chains in amphiphile from the composites. Also, the residual weight value of the composites was found to be variable compared to pristine GO sheet. Amphiphile-GO composites show almost 13.4–40.4% weight retention value at 600°C, probably due to the structural changes from different solvents and existence of carbon net-amphiphiles assembly structure in the composites [55]–[57]. Also, the present result is similar to the report about polypyrrole/graphene oxide nanocomposites [58].

**Table 2:** TGA data of GO sheet and amphiphiles-GO xerogels.

Samples	Weight loss % at temperature					Major degradation temperature (Td) °C	Weight retention (%) at 600°C
	200°C	300°C	400°C	500°C	600°C		
GO sheet	39.7	51.6	55.4	57.1	59.0	246.9	41.0
C16Py-GO (DMF)	28.1	48.7	57.2	59.8	60.5	229.2	39.5
C16Py-GO (THF)	24.8	76.0	81.8	84.5	86.6	215.1	13.4
C16Py-GO (pyridine)	19.4	59.4	64.9	67.2	68.3	219.1	31.7
BPy-GO (DMF)	34.8	57.8	70.1	73.0	73.1	233.0	26.9
BPy-GO (cyclopentanone)	11.7	45.1	55.5	57.0	59.6	222.8	40.4
BPy-GO (THF)	18.9	51.5	64.0	66.9	67.6	220.5	32.4
CTAB-GO (DMF)	17.3	54.0	61.3	66.0	68.8	229.2	31.2
CTAB-GO (cyclopentanone)	11.3	55.5	61.1	63.7	64.9	219.7	35.1
CTAB-GO (cyclohexanone)	14.2	50.1	56.9	60.8	61.3	218.1	38.7
CTAB-GO (1,4-dioxane)	9.6	51.5	56.0	57.3	59.7	217.2	40.3
CTAB-GO (THF)	20.7	60.8	68.5	72.1	72.2	228.0	27.8

**Figure 9:**
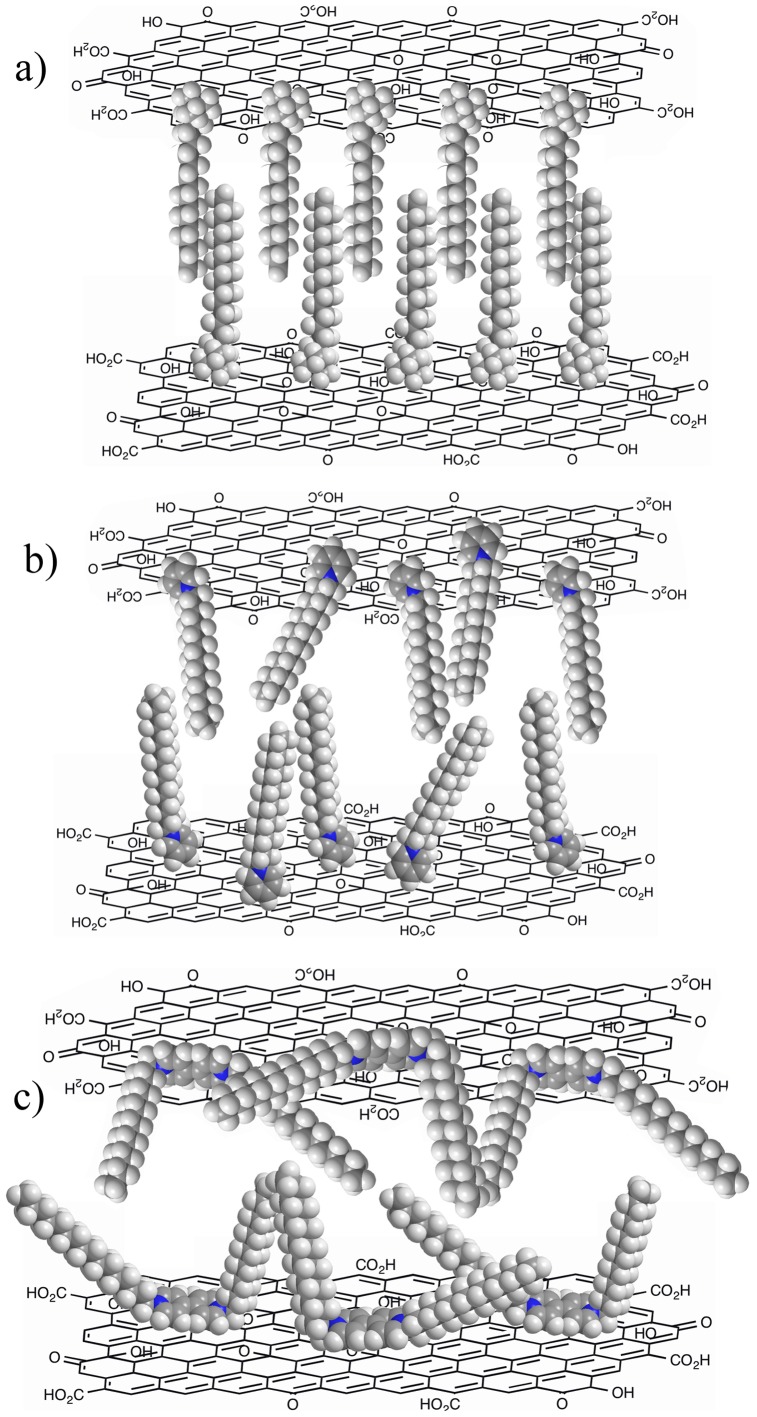
Scheme of different assembly modes in cationic amphiphiles-graphene oxide gels. CTAB-GO (a), C16Py-GO (b), and BPy-GO (c).

Considering the experimental results described above and the organized packing in the organogels, some possible assembly modes in cationic amphiphile-graphene oxide gels are proposed and schematically shown in Figure 9. As for CTAB-GO gel, due to the van der Waals force and flexibility of alkyl chains in the molecular skeleton as well as the strong electrostatic force of ammonium headgroups with oxygen-containing functional groups at GO surface, after combination with GO, orderly repeating units are obtained in different solvents. As for C16Py-GO and BPy-GO composites with additional pyridinium headgroups, the π-π stacking between carbon net in GO plane and pyridine ring appears to be competitive with the electrostatic interaction and van der Waals force. So the assembly units in nanostructures between amphiphiles and GO in present two cases are not organized sufficiently in comparison with that of CTAB-GO gel due to the combination of many kinds of forces. The present data further verify that the substituent headgroups in molecular skeletons can regulate the stacking and self-assembled nanostructures upon distinct intermolecular forces [59]–[61]. In addition, it should be noted that Drummond and co-workers have achieved a lot of excellent works relative to functionalized amphiphile self-assembly materials as prospective building blocks and investigated their self-assembly mechanisms in recent years [62]–[65]. Especially, the research work about ionic liquids as amphiphile self-assembly process focus on the nanostructure of neat ionic liquids, their solvent cohesive energy density, and the related solvophobic effect. The present experimental results showed also strong correlation with the mechanisms between nanostructure of the ionic liquid and its characteristics as an amphiphile self-assembly solvent, and could be further compared in future research work. Meanwhile, present experimental results are comparable with the results of our recent works [66], [67]. Therein, functionalized imide derivatives with the substituent groups of azobenzene and luminol residue can have a profound effect on the gelation abilities and the as-formed nanostructures of the studied compounds. For the present composite gels, the experimental data show that the headgroups in amphiphiles play a crucial role in the gelation behaviors in various organic solvents.

## Conclusions

In this study, we have demonstrated the formation of organogels by self-assembly of cationic amphiphile coupled GO composites. Their gelation behaviors in various organic solvents can be regulated by changing headgroups of amphiphiles. Ammonium headgroup of molecular skeletons in the composites is more favorable for the gelation of organic solvents in comparison with pyridinium headgroup. Headgroup effects of amphiphiles have been demonstrated to be an efficient means to manipulate self-assembly of GO-based composites. Diversity of intermolecular packing between composites and solvents is presumably responsible for presence of various nanostructures. Therefore, the present work might renew interest and provide useful exploration in the design of self-assembled GO composites and soft matters in future.
